# Supplementary material for: Reciprocal Homer1a and Homer2 Isoform Expression Is a Key Mechanism for Muscle Soleus Atrophy in Spaceflown Mice
Source: Int J Mol Sci. 2021 Dec 22;23(1):75. doi: 10.3390/ijms23010075 (PMC8744925; doi:10.3390/ijms23010075)
Supplement: Supplementary file 1 [file ijms-23-00075-s001.zip › ijms-1374574-supplementary.pdf]

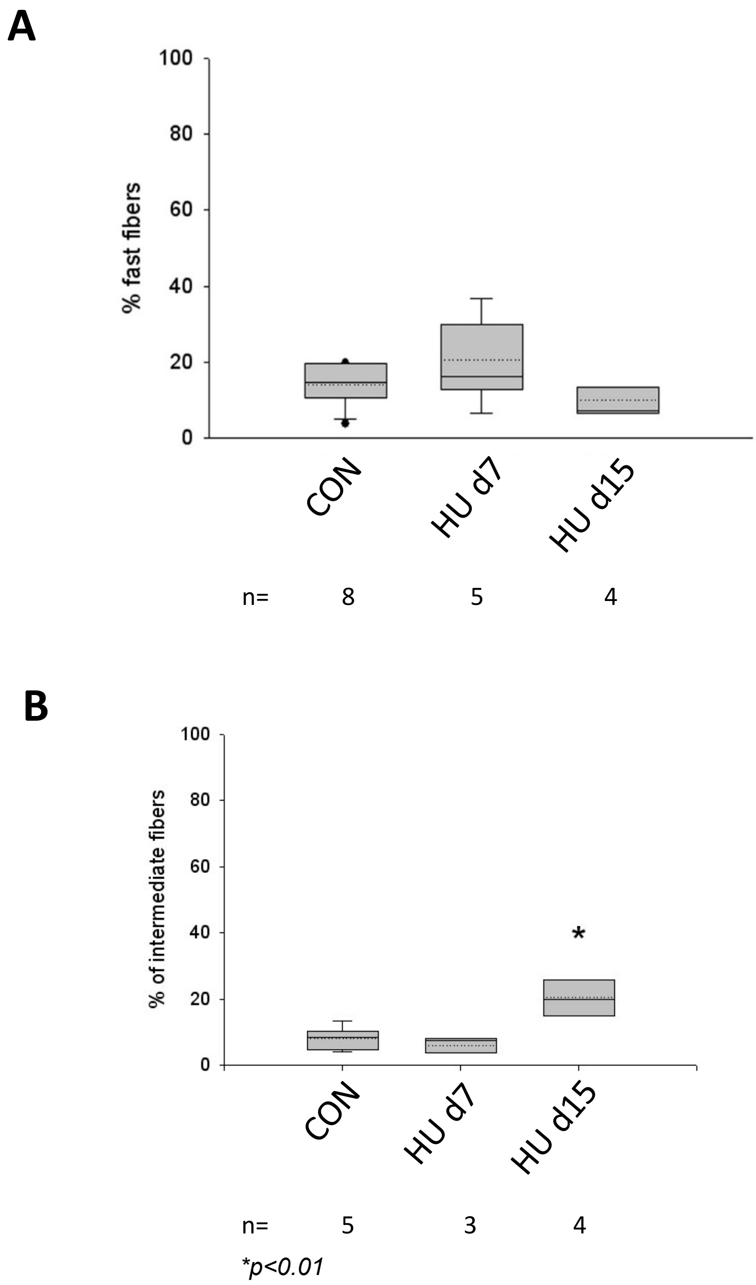

Figure S1. Myofiber phenotype composition analysis at 2-weeks of HU (rat). (**A**) Percentage of fast and (**B**) intermediate fibers, counted on serial cryosections from *SOL* muscle, obtained from CON and HU d7 and HU d15 unloaded female Wistar rats and stained either with anti-slow or anti-fast myosin heavy chain specific antibodies. Asterisk indicates the presence of significant difference (ANOVA $p < 0.01$). About 500 fibers were considered in each muscle.
